# Blood mass spectrometry detects residual disease better than standard techniques in light-chain amyloidosis

**DOI:** 10.1038/s41408-020-0291-8

**Published:** 2020-02-25

**Authors:** Angela Dispenzieri, Bonnie Arendt, Surendra Dasari, Mindy Kohlhagen, Taxiarchis Kourelis, Shaji K. Kumar, Nelson Leung, Eli Muchtar, Francis K. Buadi, Rahma Warsame, Robert A. Kyle, Martha Q. Lacy, David Dingli, Prashant Kapoor, Wilson I. Gonsalves, Ronald S. Go, Suzanne R. Hayman, Yi Lisa Hwa, Amy Fonder, Miriam Hobbs, Dragan Jevremovic, John A. Lust, Steven Zeldenrust, Steve J. Russell, S. Vincent Rajkumar, Morie A. Gertz, David Murray

**Affiliations:** 1 Department of Internal Medicine, Division of Hematology, Mayo Clinic Rochester, Rochester, MN USA; 2 Department of Laboratory Medicine and Pathology, Mayo Clinic Rochester, Rochester, MN USA; 3 Division of Nephrology and Hypertension, Mayo Clinic Rochester, Rochester, MN USA

**Keywords:** Myeloma, Myeloma

## Abstract

In patients with immunoglobulin light-chain (AL) amyloidosis, depth of hematologic response correlates with both organ response and overall survival. Our group has demonstrated that screening with a matrix-assisted laser desorption/ionization-time-of-flight (TOF) mass spectrometry (MS) is a quick, sensitive, and accurate means to diagnose and monitor the serum of patients with plasma cell disorders. Microflow liquid chromatography coupled with electrospray ionization and quadrupole TOF MS adds further sensitivity. We identified 33 patients with AL amyloidosis who achieved amyloid complete hematologic response, who also had negative bone marrow by six-color flow cytometry, and who had paired serum samples to test by MS. These samples were subjected to blood MS. Four patients (12%) were found to have residual disease by these techniques. The presence of residual disease by MS was associated with a poorer time to progression (at 50 months 75% versus 13%, *p* = 0.003). MS of the blood out-performed serum and urine immunofixation, the serum immunoglobulin free light chain, and six-color flow cytometry of the bone marrow in detecting residual disease. Additional studies that include urine MS and next-generation techniques to detect clonal plasma cells in the bone marrow will further elucidate the full potential of this technique.

## Introduction

Immunoglobulin light-chain (AL) amyloidosis is a life-threatening illness. Depth of hematologic response correlates with both organ response and overall survival. Our group has demonstrated that screening with a matrix-assisted laser desorption/ionization-time-of-flight (TOF) mass spectrometry (MASS-FIX) is a quick, inexpensive, and accurate means to diagnose and monitor the serum and urine of patients with plasma cell disorders^1–3^. Samples can be reflexed to microflow liquid chromatography coupled with electrospray ionization and quadrupole TOF mass spectrometry (ESI-TOF)^4,5^. Because these techniques provide a mass/charge for a given patient’s monoclonal protein, they can provide greater sensitivity and specificity to monitor for residual disease^5^. Our goal was to assess mass spectrometry performance in patients with AL amyloidosis who have been classified as amyloid complete hematologic response using consensus criteria^6,7^ and six-color flow cytometry of bone marrow.

## Methods

The Mayo Foundation Institutional Review Board (IRB) approved the study. All patients gave written informed consent to have their medical records reviewed and samples analyzed according to IRB requirements and federal regulations. Patients were eligible for this retrospective study if they: (1) were diagnosed with AL amyloidosis between January 2000 and May 2015; (2) were classified as amyloidosis complete hematologic response by immunofixation electrophoresis (IFE), serum free light chain (FLC) by consensus criteria;^6,7^ (3) had a negative bone marrow by six-color flow cytometry; and (4) had both a stored research sample prior to starting a line of therapy and a repeat sample while in complete hematologic response. The diagnosis of amyloidosis was made by Congo red with green birefringence under polarized light; the typing of the amyloid was with immunohistochemical stains or proteomics^8,9^. Supplementary Figure 1 is a consort diagram illustrating patient selection. Median time from institution of therapy to complete response (CR) sample was 18 months (interquartile range 9.1, 20 months).

The serum IFE (SIFE), urine IFE (UIFE), FLC, and bone marrow measurements were done as part of routine clinical practice as previously described^4,5^. Urine samples were concentrated to a maximum of 200× to achieve final concentrations of urine protein between 20 and 80 g/L^4,5^. The FLC assay (Freelite™, The Binding Site Ltd.) was performed on a Siemens BNII nephelometer^10^, and an abnormal FLC result was defined as an abnormal FLC κ/λ ratio. Bone marrow clonality was determined by six-color flow cytometry^11^. This method has sensitivity of ~10^−4^ to 10^−5^.

For MASS-FIX, immunoglobulins were enriched from serum using camelid-derived nanobodies directed against the heavy-chain constant domains of IgG, IgA, and IgM or the light-chain constant domains of κ and λ (Thermo Fisher Scientific)^4,5^. The +1 and +2 charge states of the light chains and heavy chains were measured by configuring the mass spectrometer to analyze ions between an *m*/*z* of 9000–32,000 Da.

Samples were subjected to an additional liquid chromatography electrospray ionization tandem mass spectrometric analysis on a Q-TOF mass spectrometer [SCIEX TripleTOF 5600 quadrupole MS (Vaughan, ON, Canada) operating in ESI-positive mode with a Turbo V dual ion source with an automated calibrant delivery system]^12^.

Resulting mass spectra of each sample were visually inspected by three independent reviewers (D.M., B.A., and A.D.), in order to detect and isotype any M proteins present in the patient. All reviewers were blinded to clinical information at the time of the spectral analysis. Clinical histories were reviewed by A.D.

Hematologic progression was defined according to the consensus criteria^6,7^ with the addition of including commencement of a new line of clone-directed therapy based on worsening of hematologic parameters as progression. Death, in the absence of hematologic progression, was not considered to be a progression. Overall survival and time to progression were calculated according the methods of Kaplan–Meier and differences were determined by Wilcoxon. Statistics were performed using JMP PRO 14.1.0 (SAS, NC).

## Results

Thirty-three patients met the criteria of CR by blood and bone marrow, and their baseline characteristics are shown in Table 1. Median age was 56 years. Fifty-five percent were male, and all were Caucasian. No test performed perfectly at baseline with the exception of the MASS-FIX due to inclusion requirements. The positive baseline results for the other assays are shown in Supplementary Fig. 2: SIFE, 85%; UIFE, 79%; and abnormal FLC ratio, 84%. Five SIFE-negative patients were positive by MASS-FIX and ESI-TOF, another SIFE negative was found to have a monoclonal λ by ESI-TOF and UIFE, and four SIFE negatives had abnormal FLC ratios. Apart from disagreements between positive and negative, isotype discrepancies were seen between SIFE and MASS-FIX in only one instance: IgG λ by SIFE, but free λ by MASS-FIX; ESI-TOF detected the IgG λ. No isotype discrepancies were observed between FLC and MASS-FIX apart from the disagreements in four cases in which MASS-FIX was positive and FLC negative. There were eight disagreements in positive–negative calls between FLC and SIFE.

**Table 1:** On-study and treatment characteristics.

	All patients (*n* = 33)
Age (years), median (range)	56 (44, 81)
Male gender, *n* (%)	18 (55)
Creatinine (mg/dL), median (range)	1.1 (0.7, 5.4)
Creatinine ≥2 mg/dL, *n* (%)	3 (9)
iFLC (mg/dL), median (range)	13.2 (2.0, 195)
Abnormal FLC ratio^a,b^, *n* (%)	27 (84)
SIFE positive, *n* (%)	28 (85)
SIFE HC^c^, G/A/D/Neg, *n*	12/9/1/12
MALDI positive, *n* (%)	33 (100)
UIFE positive, *n* (%)	23^d^ (79)
BMPC (%) (range)	9 (2, 40)
Amyloid type: κ/λ, *n* (%)	5/28 (15/85)
Mayo stage 2004^b^, I, II, IIIa /IIIb, *n*	12/11/8/0
Mayo stage 2012^b^, I, II, III, IV, *n*	13/8/4/6
Organ involvement, H, K, L, O, *n*	10/25 /1/4
Line of therapy	
First line	31 (93)
Second line	2 (7)
Therapies	
ASCT, no induction	19 (57)
ASCT induction	9 (27)
Mel-Dex	2 (6)
CVD	2 (6)
CRD	1 (3)

*H* heart, *K* kidney, *L* liver, *O*, other which includes GI, nerve, and lung.

^a^Outside normal range, which is 0.26 and 1.65 mg/dL.

^b^One missing data.

^c^One biclonal patient (L + GK) counted twice.

^d^Four missing data.

**Fig. 1:**
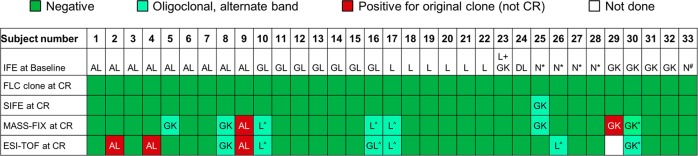
Grading of complete response using various techniques. Any SIFE with a subsequent isotype discordant with baseline represents oligoclonal banding and unrelated to the original clone. *L by MASS-FIX; ^#^AK by MASS-FIX; ^different mass, so considered negative.

**Fig. 2:**
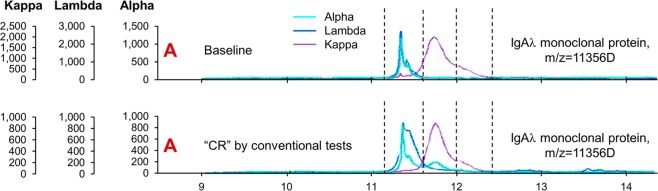
Example of positive MASS-FIX in patient otherwise deemed to be in complete response.

At CR assessment, by definition all patients had negative SIFE, negative UIFE, normal FLC ratio, and a negative bone marrow by six-color flow cytometry. By MASS-FIX and ESI-TOF, respectively, two and four patients were found to have their original monoclonal protein detected at CR determination (Figs. 1 and 2). Another eight had monoclonal proteins that did not coincide with their original protein at CR measurement, consistent with transient post-therapy oligoclonal banding. Therefore, a total 12% (4 of 33) of patients who were thought to be in CR by high-resolution bone marrow flow cytometry, SIFE, UIFE, and FLC were found to have residual disease by mass spectrometric techniques of the blood.

**Fig. 3:**
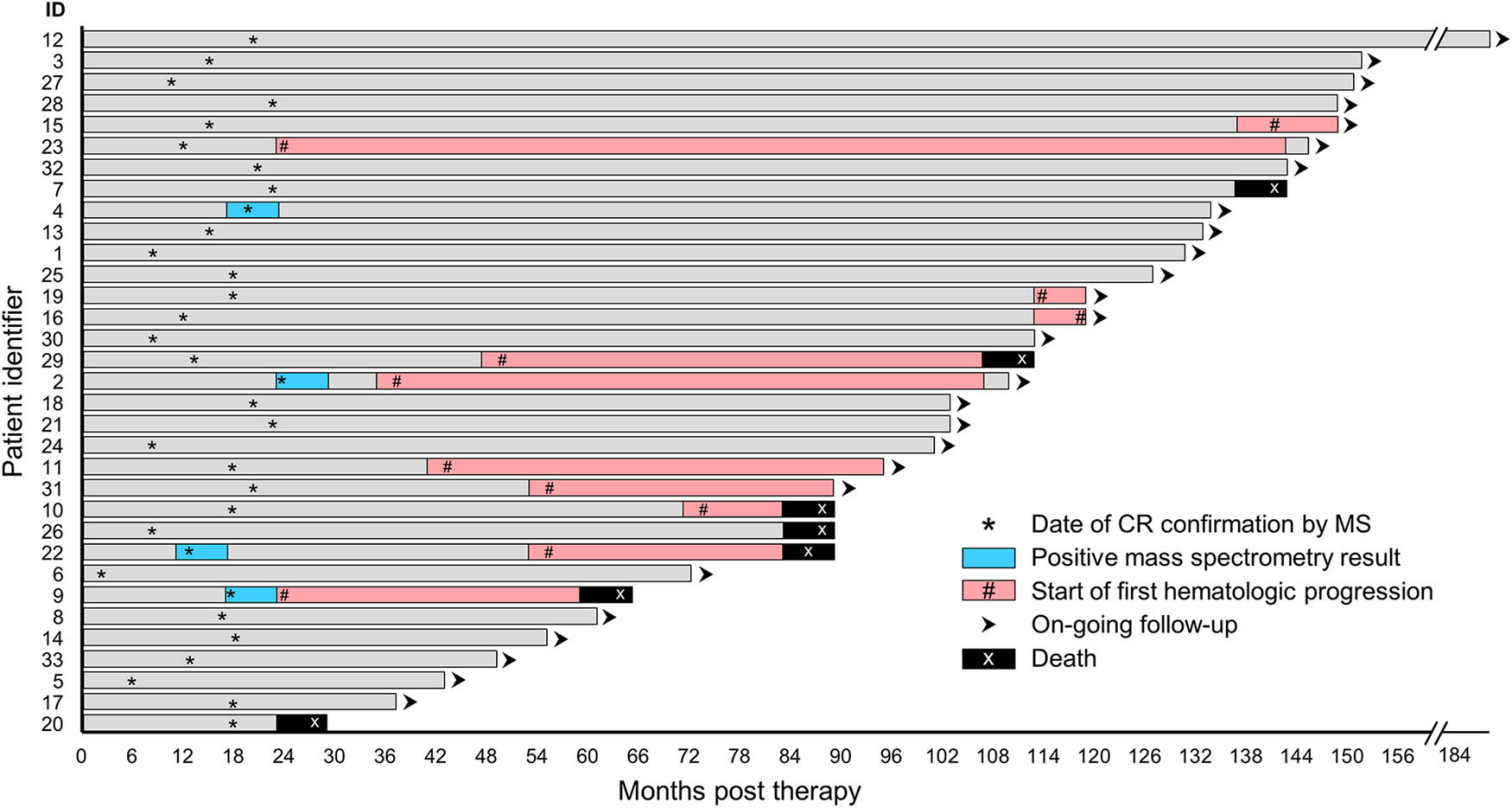
Patient outcomes details.

**Fig. 4:**
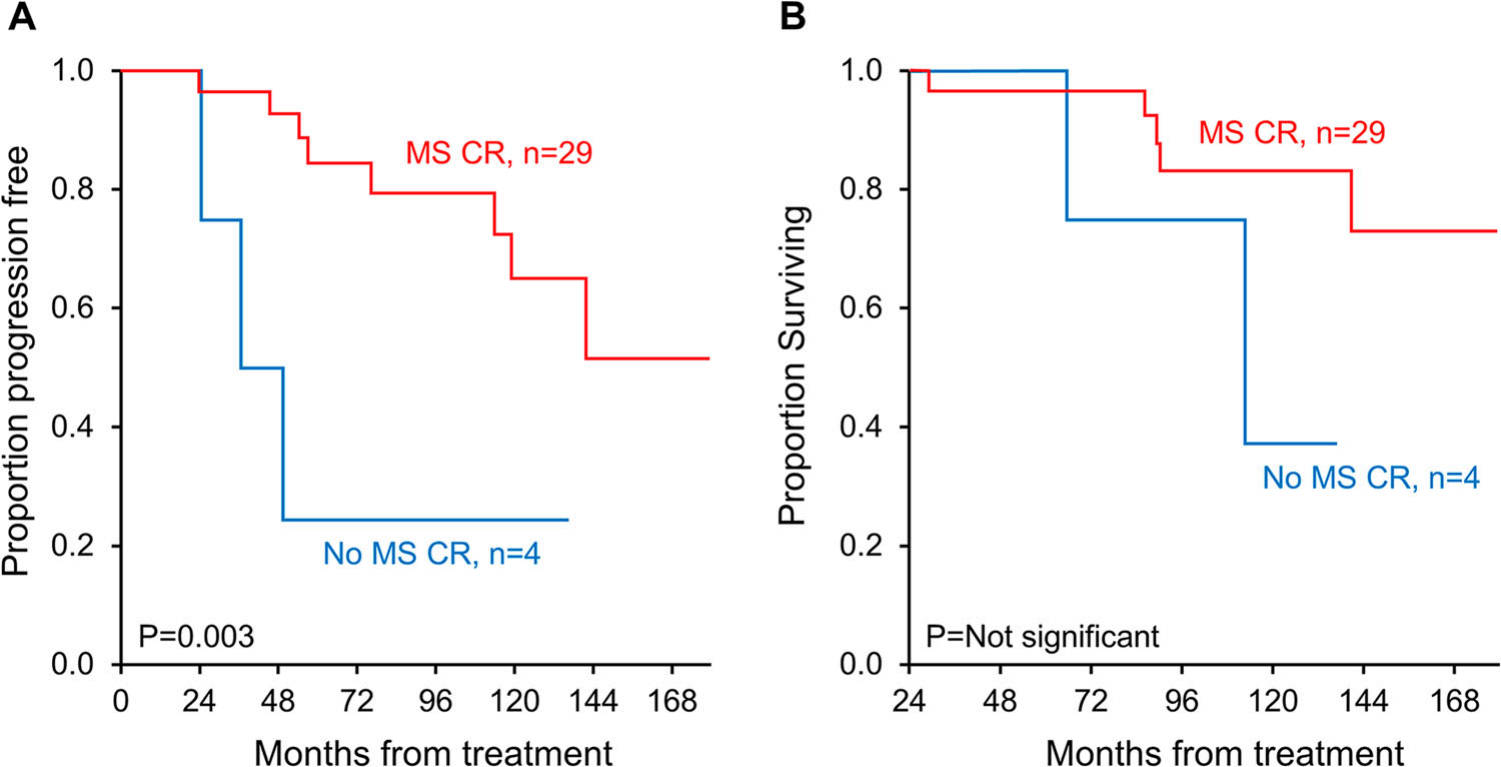
Survival outcomes when using mass spectrometry. **a** Time to progression based on mass spectrometry analysis (either MASS-FIX or ESI-TOF) was positive. **b** Overall survival based on mass spectrometry analysis (either MASS-FIX or ESI-TOF) was positive.

We next evaluated the effect of a positive result by mass spectrometry on time to progression and overall survival (Fig. 3). Median follow-up for the cohort was 116 months (range 37, 183 months). In the mass spectrometry-positive group, by 50 months 75% of patients had progression events in contrast to 13% in the mass spectrometry-negative group, *p* = 0.003 (Fig. 4a). Respective 10-year overall survival rates were 62% and 83%, *p* = not significant (Fig. 4b). Twenty-two patients achieved organ response, seven did not, and four were not accessible due to non-measurable disease (two gastrointestinal, one pulmonary, and one nerve). Of the four positive mass spectrometry patients, there were two who had organ response, one who did not, and another who did not have measurable disease (pulmonary disease only).

## Discussion

We have demonstrated the value of MASS-FIX in detecting residual disease in patients with AL amyloidosis. MS detected residual disease among AL amyloidosis patients in hematologic CR, not only in the context of negative blood and urine IFE and serum FLC but also in the context of a negative bone marrow employing six-color flow cytometry, which has approximately one order of magnitude less sensitivity than the next-generation flow cytometry and two orders of magnitude than the next-generation sequencing. Current consensus response criteria exclude bone marrow as part of hematologic response^6,7^, but we included bone marrow response because emerging data demonstrate that patients with a negative bone marrow by flow cytometry fare better than those without^13–18^. Despite the limited sample size, there was a very significant difference in progression-free survival between the MS-positive and -negative patients. Although there was a trend in better overall survival in the MS-negative patients, the study was likely underpowered to be significant.

Prior studies in AL amyloidosis and other plasma cell disorders have demonstrated the higher sensitivity and specificity of this assay^2,5,19^. A similar study of myeloma patients in stringent CR showed greater sensitivity for these mass spectrometry techniques^1^. One would anticipate that the overall performance of the mass spectrometry approach would have been even better had there also been urine samples to test by mass spectrometry^1,2^.

Additional work in larger numbers of patients will ultimately be required to determine the range of sensitivity of mass spectroscopy of blood and urine has relative to the next-generation bone marrow testing, but these results are very promising.

## Supplementary information

Supplementary figures
