# Supplementary figures and images for: Blood mass spectrometry detects residual disease better than standard techniques in light-chain amyloidosis
Source: Blood Cancer J. 2020 Feb 25;10(2):20. doi: 10.1038/s41408-020-0291-8 (PMC7042300; doi:10.1038/s41408-020-0291-8)

## Slide 1
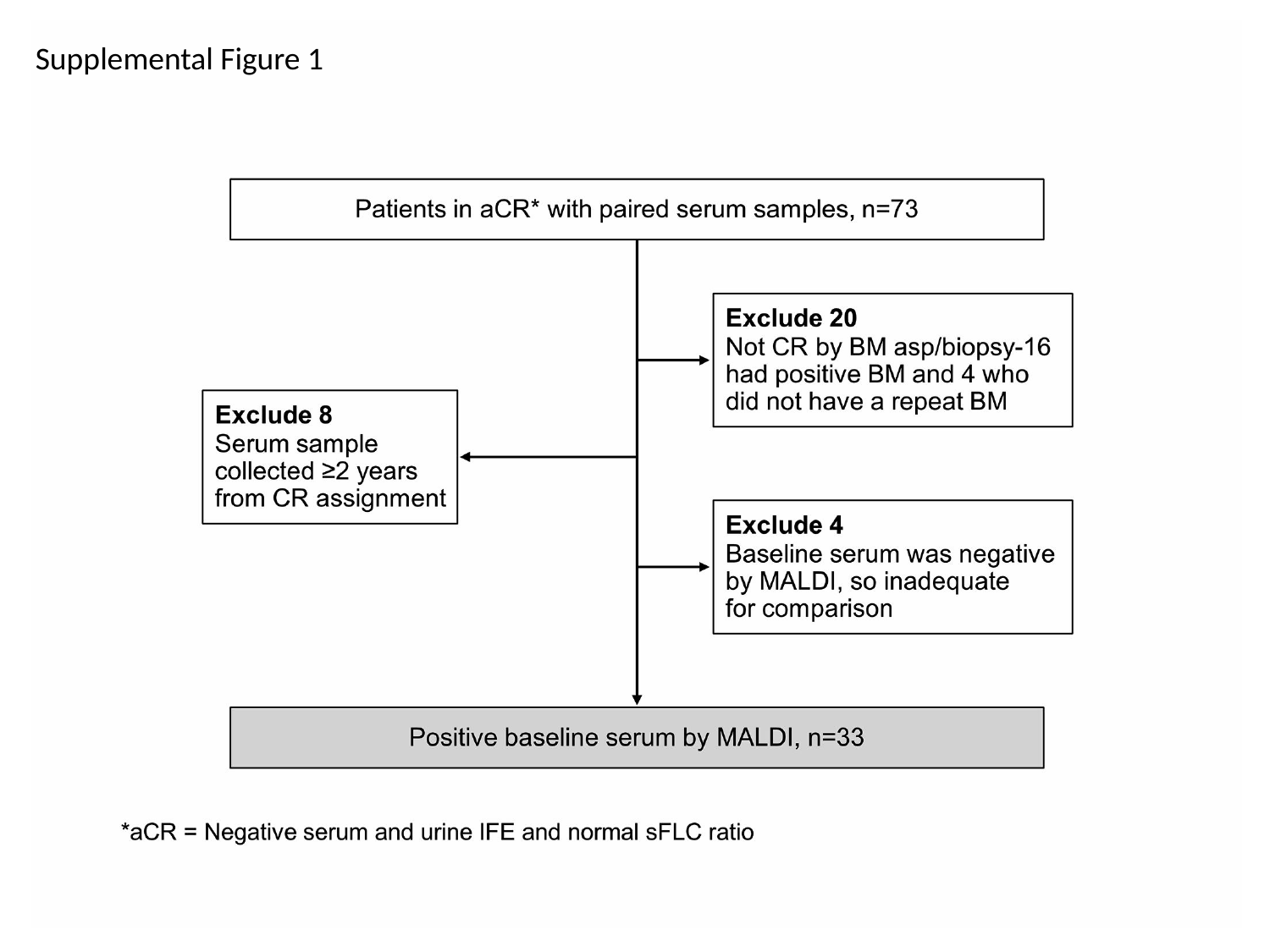

Supplemental Figure 1

## Slide 2
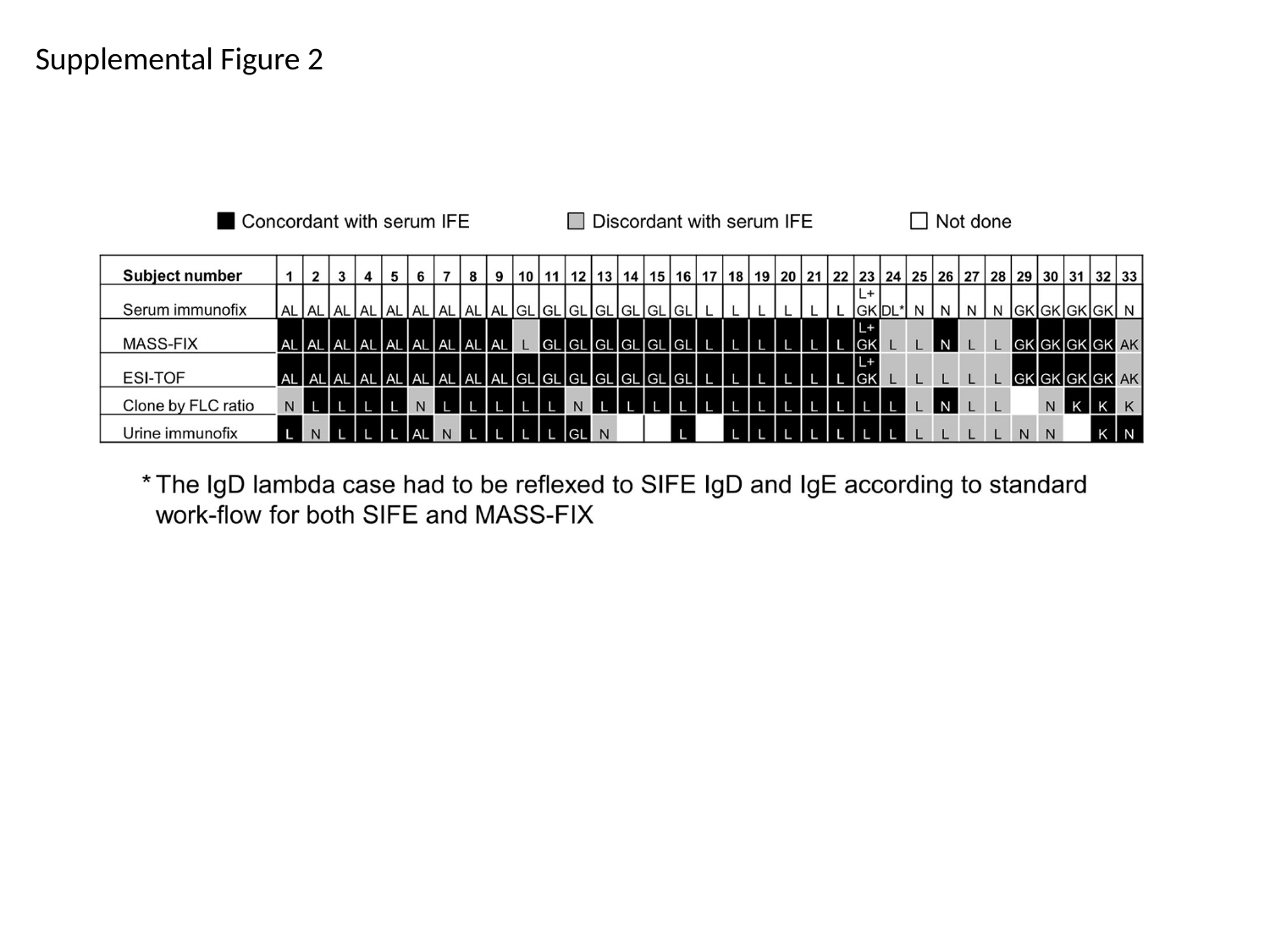

Supplemental Figure 2

## Slide 3
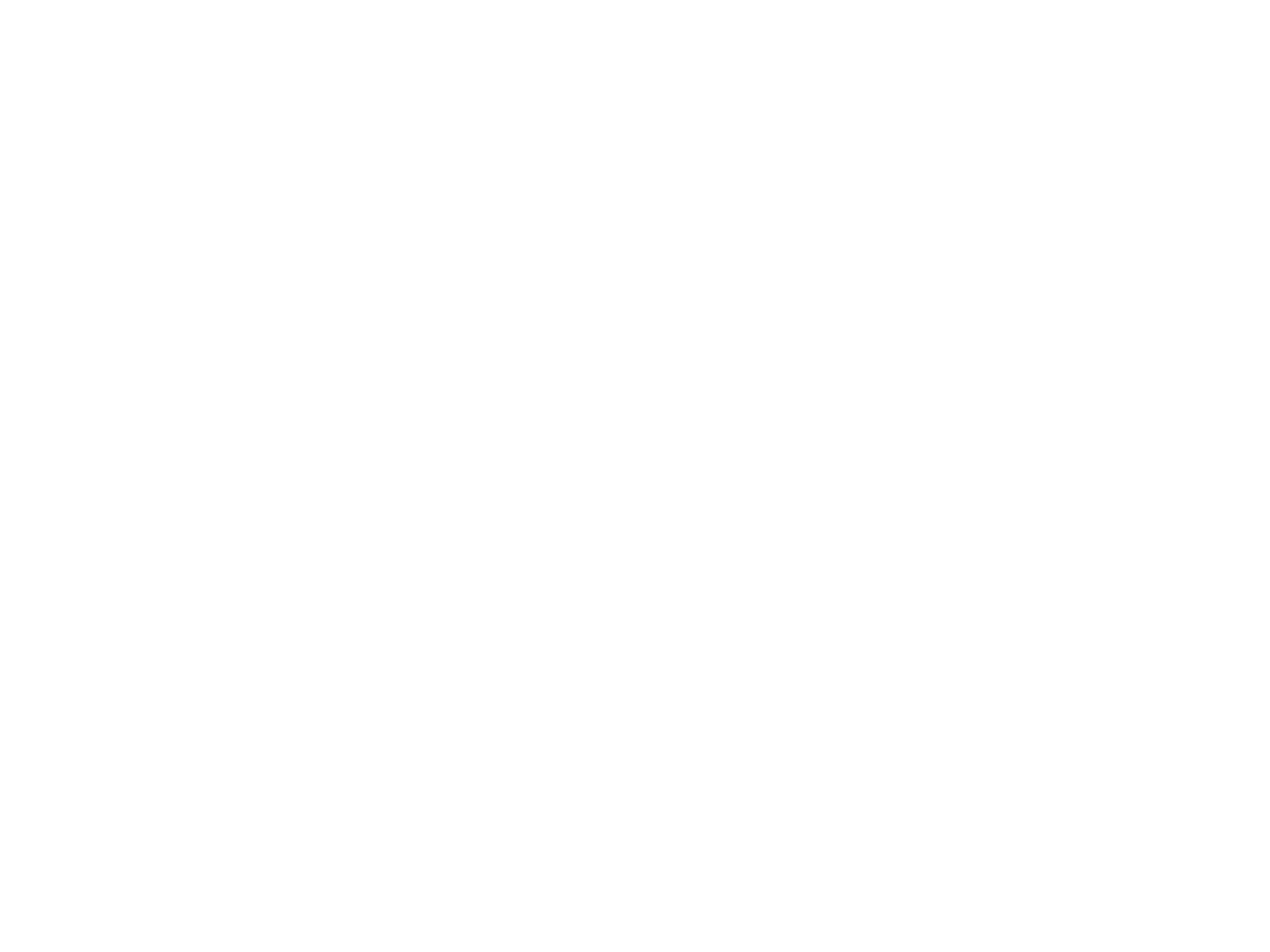

## Slide 4
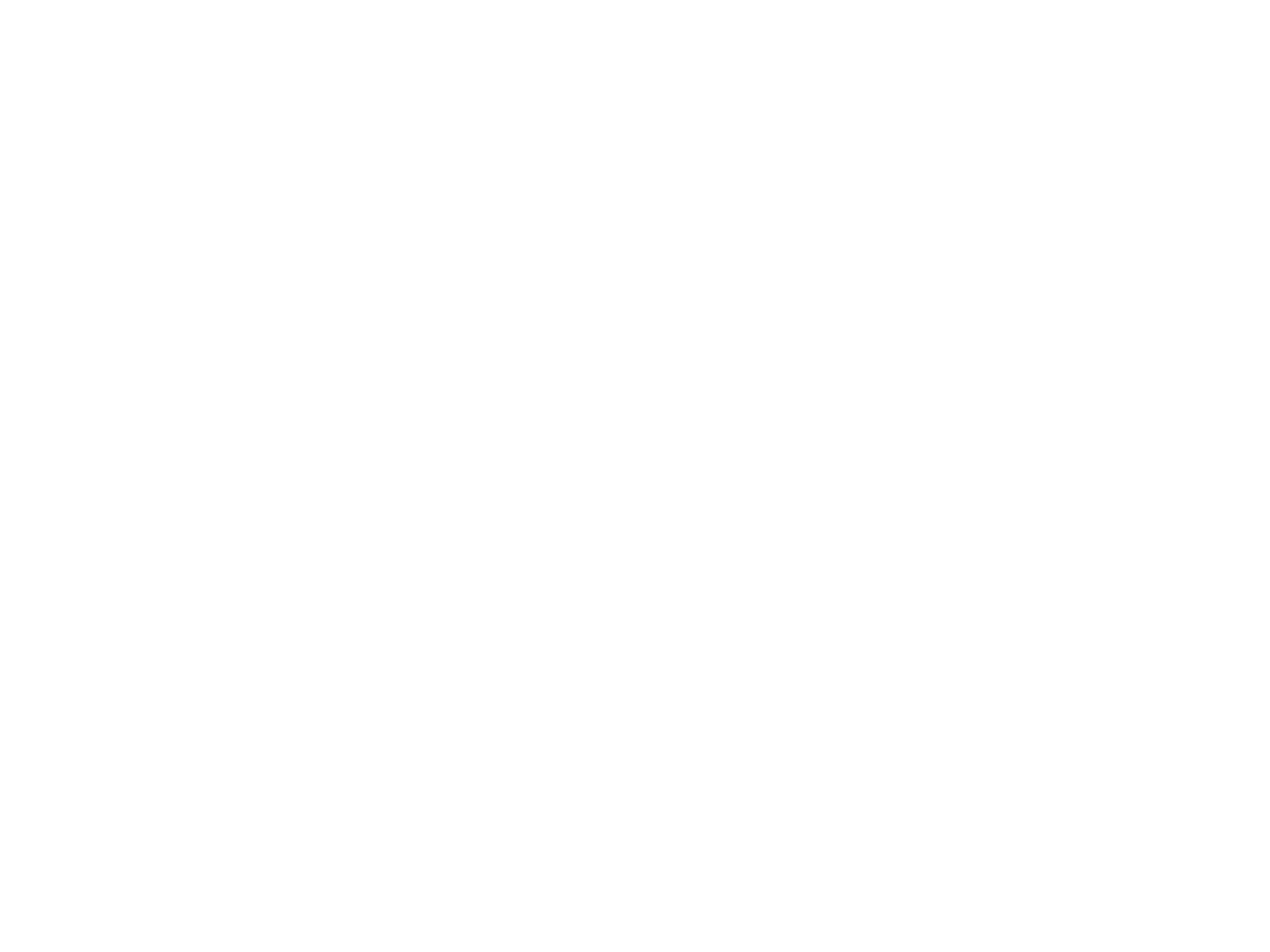

Supplement: Supplementary file 1 — Supplementary figures [file 41408_2020_291_MOESM1_ESM.pptx]
